# Supplementary material for: Exogenous fatty acids inhibit fatty acid synthesis by competing with endogenously generated substrates for phospholipid synthesis in Escherichia coli
Source: FEBS Lett. 2024 Dec 30;599(5):667–81. doi: 10.1002/1873-3468.15092 (PMC11891403; doi:10.1002/1873-3468.15092)
Supplement: Supplementary file 1 — Fig. S1. Phospholipid abundance from initiation of the simulation to the stepwise increase of acyl‐CoA at 8000 s. Table S1. Initial concentrations of species set at non‐zero values. Table S2. Reaction parameters used. [file FEB2-599-667-s001.pdf]

Supplementary Information

Exogenous fatty acids inhibit fatty acid synthesis by competing with endogenously generated substrates for phospholipid synthesis in *Escherichia coli*

Stefan Pieter Hendrik van den Berg[1], Adja Zoumaro-Djayoon, Flora Yang, Gregory Bokinsky

Department of Bionanoscience, Kavli Institute of Nanoscience, Delft University of Technology, Delft, The Netherlands.

[1] Current affiliation: Department of Immunopathology, Sanquin Research Amsterdam, Amsterdam, The Netherlands

Materials and correspondence: g.e.bokinsky@tudelft.nl

Contents

Model description....................2

COPASI settings.....................2

Initiation (ACC-FabD)....................2

Initiation (FabH)....................3

Acyl-ACP elongation ("FabB")....................3

Substrate competition for PlsB and LPA synthesis....................3

Substrate competition for PlsC and PA synthesis....................4

Phospholipid synthesis and phospholipid dilution by simulated growth....................5

Supplemental References....................7

Model description

COPASI settings.

COPASI version 4.39 was used for all simulations. The model topology is depicted in Figure 4 in the main text. All data were generated using the "Time Course" Task, simulating 15000 seconds in 5 second intervals with deterministic (LSODA) method with default parameters (relative tolerance 10^{-6}, absolute tolerance 10^{-12}, 100000 maximum internal steps, 0 maximum internal step size). Concentrations of all species (except species listed in **Table S1**) were initialized at 0 μM. All concentrations were subsequently allowed to evolve as the simulation progressed unless fixed at set values (indicated in **Table S1**). The simulation was initiated with acyl-CoA set at zero to allow pools of acyl-ACP, phospholipid synthesis intermediates, and phospholipid abundance to reach steady-state from initial conditions. At 8000 seconds, the "Events" function triggered a stepwise increase of acyl-CoA concentration from 0 to 5 μM.

Table S1. Initial concentrations of species set at non-zero values.

Acetyl-CoA	600 μM	fixed
Holo-ACP	50 μM	Consumed/regenerated by reactions
FabH	1.6 μM	fixed
FabB	5 μM	fixed
PlsB	0.45 μM	Consumed /regenerated by substrate binding and reactions
PlsC	0.4 μM	Consumed /regenerated by substrate binding and reactions
CdsA	0.2 μM	fixed
Acyl-CoA	0 μM	Fixed, then stepwise change to fixed value (5 μM) to simulate feeding from a large pool of exogenous fatty acids

Parameters for all equations described below are given in **Table S2.**

Initiation (ACC-FabD)

The rate of fatty acid synthesis initiation from acetyl-CoA v_{ACC} was modelled using a combined ACC-FadD reaction that generates malonyl-ACP from holo-ACP and acetyl-CoA. The rate law used was based on an irreversible bimolecular reaction and included an empirical inhibition term **(Equation S1)**. The concentration of the inhibitor pool (long-chain acyl-ACP) was defined as the sum of free C16:0, C18:0, C16:1, C18:1, and C20:1 ACP concentrations. Acyl-ACP bound within an enzyme-substrate complex with PlsB or PlsC was not included in this term.

(Eq. S1) $$v_{\text{ACC}} = \frac{Vmax*[\text{acetyl-CoA}]*[\text{holo-ACP}]}{Kma*Kmb+[\text{acetyl-CoA}]*Kmb+[\text{holo-ACP}]*Kma+[\text{acetyl-CoA}]*[\text{holo-ACP}]} * \left(\frac{Ki}{Ki+[\text{long chain acyl-ACP}]}\right)$$

Initiation (FabH)

The FabH reaction was modelled using a simple irreversible bimolecular rate law (v_{FabH}) that generates C4:0 ACP from acetyl-CoA and malonyl-ACP **(Equation S2)**.

(Eq. S2)
$$v_{\text{FabH}} = \frac{k_{cat} * [\text{FabH}] * [\text{acetyl-CoA}] * [\text{malonyl-ACP}]}{Kma * Kmb + [\text{acetyl-CoA}] * Kmb + [\text{malonyl-ACP}] * Kma + [\text{acetyl-CoA}] * [\text{malonyl-ACP}]}$$

Acyl-ACP elongation ("FabB")

All elongation reactions after FabH were labelled "FabB" (combining FabB and FabF reactions) and were modelled using a simple irreversible bimolecular rate law v_{FabB} that uses malonyl-ACP and acyl-ACP of length *n* to generate acyl-ACP of length *n*+2 **(Equation S3)**. To account for the different activities of FabB and FabF on acyl-ACP chains of different length and saturation, a coefficient *c*(*n*) was applied for each chain length. These coefficients were chosen based on experimentally-determined chain length-dependent activities of FabB and FabF (1).

For the branch point between saturated and unsaturated synthesis, two parallel reactions consuming C10:0 ACP were implemented that produced either C12:0 or C12:1 ACP.

(Eq. S3)
$$v_{\text{FabB}} = \frac{c(n) * k_{cat} * [\text{FabB}] * [\text{acyl-ACP}(n)] * [\text{malonyl-ACP}]}{Kma * Kmb + [\text{acyl-ACP}] * Kmb + [\text{malonyl-ACP}] * Kma + [\text{acyl-ACP}] * [\text{malonyl-ACP}]}$$

Substrate competition for PlsB and LPA synthesis.

To simulate substrate competition between long-chain acyl-CoA and acyl-ACP, all binding and release steps involving acyl-thioester substrates with PlsB were explicitly included as separate reactions **(Scheme S1)**. This approach takes inspiration from a complete model of the *E. coli* fatty acid synthesis pathway (2). All PlsB-substrate complexes reacted at identical rates, generating free PlsB, the corresponding LPA depending on the acyl chain transferred, and holo-ACP if an acyl-ACP thioester was used as a substrate (free CoA concentrations were not included in the model, so CoA generation from reactions with acyl-CoA is not accounted for). The PlsB substrate glycerol-3-phosphate (G3P) was also not explicitly included (equivalent to setting G3P at a fixed concentration).

$$\text{acyl-thioester + PlsB} \underset{k_{\text{off}}}{\overset{k_{\text{on}}}{\rightleftharpoons}} \text{PlsB-substrate complex} \xrightarrow{k_{\text{cat}} * f([\text{total PL}])} \text{PlsB + LPA (+ holo-ACP)}$$

(Scheme S1)

In *E. coli,* the phospholipid/protein ratio is stringently maintained. This means that neither phospholipid abundance nor the rate of phospholipid synthesis is affected by increased long-chain acyl-thioester concentrations caused by conversion of exogenous fatty acids to acyl-CoA. To stabilize phospholipid abundance, we created an inhibition function *f*([total PL]) that reduces the

LPA synthesis reaction rate according to the abundance of total phospholipids accumulated **(Equation S4)**:

(Eq. S4)

$$f([\text{total PL}]) = \frac{1}{1+\frac{[\text{total PL}]}{Ki}}$$

This introduces feedback inhibition of PlsB by phospholipid accumulation. The feedback inhibition effectively stabilizes flux through PlsB despite the stepwise increase in acyl-CoA **(Figure S1)**.

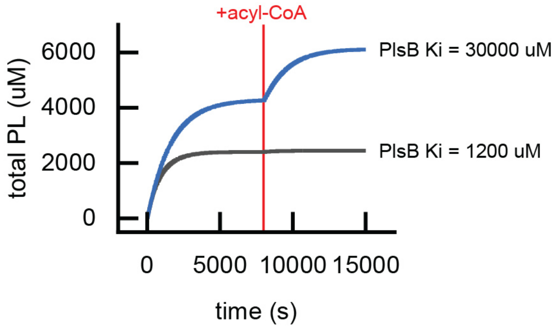

Figure S1. Phospholipid abundance from initiation of the simulation to the stepwise increase of acyl-CoA at 8000 s. Implementing feedback inhibition on PlsB activity by total phospholipid content stabilizes phospholipid abundance despite the increase of all substrates triggered by acyl-CoA (depicted by black line generated from setting the PlsB inhibition constant in **Equation S4** to 1200 μM). Removing feedback by increasing the inhibition term K_I to 30000 μM (blue line) allows acyl-CoA synthesis to increase phospholipid abundance.

In the model, PlsB binds and reacts with C16:0, C18:0, C16:1, and C18:1 thioesters; however PlsB does not react efficiently with C16:1 thioesters (3). To implement substrate preferences for PlsB, a coefficient was introduced for the C16:1 thioester binding constant. All PlsB-substrate complexes reacted at identical rates.

Substrate competition for PlsC and PA synthesis.

Acyl-thioester substrate competition for PlsC was implemented similarly as for PlsB **(Scheme S2)**. In the model, PlsC binds and reacts with C16:0, C16:1, C18:1, and C20:1 thioesters but does not react efficiently with C16:0 thioesters. Thus a coefficient was introduced for the binding constant for C16:0 thioester species to reduce substrate binding relative to preferred substrates.

$$\text{acyl-thioester + PlsC} \underset{k_{\text{off}}}{\overset{k_{\text{on}}}{\rightleftharpoons}} \text{PlsC-substrate complex + LPA} \xrightarrow{v_{\text{PlsC}}} \text{PlsC + PA (+ holo-ACP)}$$

(Scheme S2)

All PlsC-acyl-thioester enzyme-substrate complex subsequently reacted with each individual LPA species according to a standard Michaelis-Menten kinetic function v_{PlsC} **(Equation S5)**:

(Eq. S5) $$v_{\mathrm{PlsC}} = \frac{k_{cat} * [\text{PlsC-substrate complex}] * [\mathrm{LPA}]}{K_M + [\mathrm{LPA}]}$$

Phospholipid synthesis and phospholipid dilution by simulated growth.

For simplicity, conversion of each individual PA species to its corresponding membrane phospholipid was simulated as a single step labelled as "CdsA". This combines PA conversion to CDP-DAG by CdsA and all subsequent pathways for generation of PG and PE. The reaction rate v_{PL} converting each individual PA species to its corresponding phospholipid was simulated as a single-substrate Michaelis-Menten reaction **(Equation S6)**:

(Eq. S6) $$v_{\mathrm{PL}} = \frac{k_{cat} * [\mathrm{CdsA}] * [\mathrm{PA}]}{K_M + [\mathrm{PA}]}$$

To record acyl chain distributions in simulated membrane phospholipids and account for total phospholipid abundance (which allows its use as a feedback inhibition signal controlling PlsB), phospholipids produced by the "CdsA" reaction with PA were steadily depleted by the model according to simple mass action kinetics **(Equation S7)**:

(Eq. S7) $$v_{\mathrm{PL\ dilution}} = k_{growth} * [\mathrm{PL}]$$

**Table S2.** Reaction parameters used.	
**ACC-FabD initiation (Equation S1)**	
Vmax	100 μM/sec
Kma (for acetyl-CoA)	500 μM
Kmb (for holo-ACP)	10 μM
Ki	5 μM
**FabH (Equation S2)**	
kcat	5/sec
[FabH]	1.6 μM
Kma (for acetyl-CoA)	10 μM
Kmb (for malonyl-ACP)	2 μM
**FabB (Equation S3)**	
kcat	10/sec
[FabB]	5 μM
Kma (for acyl-ACP)	2.5 μM
KmB (for malonyl-ACP)	2.5 μM
c(C4:0-ACP)	0.33
c(C6:0-ACP)	0.86
c(C8:0-ACP)	0.97
c(C10:0-ACP) to C12:0-ACP (saturated branch)	0.97
c(C10:0-ACP) to C12:1-ACP (unsaturated branch)	2.5*c(C10:0-ACP)
c(C12:0-ACP)	1.0
c(C14:0-ACP)	0.3
c(C16:0-ACP)	0.02
c(C12:1-ACP)	1.0
c(C14:1-ACP)	0.3
c(C16:1-ACP)	0.15
c(C18:1-ACP)	0.01
**PlsB substrate binding, catalysis, and inhibition (Scheme S1, Equation S4)**	
kon	1000/(μM*s)
koff	100/s
kon(16:1)	0.05*kon
kcat	10/s
Ki	1200 μM
**PlsC substrate binding and catalysis (Scheme S2, Equation S5)**	
kon	1000/(μM*s)
koff	100/s
kon(16:0)	0.05*kon
kcat	10/s
Km	0.15 μM
**CdsA (phospholipid synthesis from PA) (Equation S6)**	
kcat	10/s
[CdsA]	0.2 μM
Km	10 μM
**Phospholipid dilution by growth (Equation S7)**	
kgrowth	0.0006/s

Supplemental References

1. P. Edwards, J. S. Nelsen, J. G. Metz, K. Dehesh, Cloning of the fabF gene in an expression vector and in vitro characterization of recombinant fabF and fabB encoded enzymes from Escherichia coli. *FEBS Lett.* **402**, 62–66 (1997).
2. S. Ruppe, K. Mains, J. M. Fox, A kinetic rationale for functional redundancy in fatty acid biosynthesis. *Proc. Natl. Acad. Sci. U. S. A.* **117**, 23557–23564 (2020).
3. L. Hoogerland, *et al.*, A temperature-sensitive metabolic valve and a transcriptional feedback loop drive rapid homeoviscous adaptation in Escherichia coli. *Nat. Commun.* **15**, 9386 (2024).
